# A french adaptation of the vineland adaptive behavior scales VABS-II

**DOI:** 10.1192/j.eurpsy.2021.421

**Published:** 2021-08-13

**Authors:** N. Touil, B. Riche, V. Des Portes, S. Mardirossian, S. Gaillard, M. Rabilloud, B. Kassai, S. Sonie

**Affiliations:** 1 Centre De Ressources Autisme Rhône Alpes, CH LE VINATIER, BRON, France; 2 Service De Biostatistiques, HOSPICES CIVILS DE LYON, Lyon, France; 3 Service De Neurologie Pédiatrique, HOSPICES CIVILS DE LYON, Bron, France; 4 Epicime-cic 1407 De Lyon, HOSPICES CIVILS DE LYON, BRON, France

**Keywords:** autism spectrum disorder, French norms, adaptative behavior, validation

## Abstract

**Introduction:**

For populations with intellectual disability and autism spectrum disorder, it is essential to complete cognitive assessment with an adaptive behavior scale.

**Objectives:**

To translate VABS-II from English to French and establish norms for the French population.

**Methods:**

We used the Parent/Caregiver form in order to assess psychometric characteristics of the VABS-II and to develop norms for the French population. VABS-II comprises 4 domains, 11 subdomains, and an optional maladaptive behavior index. The French translation of the VABS-II followed standard cross-cultural translation methods. The study was performed in the Rhône-Alpes-Auvergne department comprising 12 % of France inhabitants and well representing the French general population.

**Results:**

From 4576 VABS-II questionnaires distributed, 1707 were returned and 1654 were analyzed. The reason for exclusion was the impossibility to score one of the subdomains. From 174 questionnaires included in the test-retest, 95 were analyzed, and 79 questionnaires were excluded because 86 under 34 days, 8 > 3 months, and one participant that changed age group between test and retest. Scores based on French norms fluctuated around values based on US norms on all subdomains.

**Conclusions:**

The French Vineland questionnaire is the single test with the adequate norms to allow identifying children with adaptive behavior difficulties. It should be used as a complement of the assessment of the intellectual quotient, according to DSM V, for the diagnosis of intellectual disabilities. It must be done in reference to the developmental and cultural standards specific to the environment in which the person is evolving.

**Disclosure:**

No significant relationships.

